# Anisotropic distortion in the perceived direction of motion on the arm

**DOI:** 10.1038/s41598-022-27032-x

**Published:** 2023-01-02

**Authors:** Scinob Kuroki

**Affiliations:** NTT Communication Science Laboratories, NTT Corporation, 3-1, Morinosato-Wakamiya, Atsugi, Kanagawa, 2430198 Japan

**Keywords:** Human behaviour, Somatosensory system, Sensory processing

## Abstract

Skin covers the entire body, and its thickness and distribution of mechanoreceptors vary markedly across body parts. It has been shown that the brain is not able to fully compensate for such anisotropy, and as a result, the representational space of touch differs depending on which parts the stimulus is applied to. Here, by contrasting the hand and arm, we investigated the difference in perceived motion. Using a large-area braille display, we were able to present precisely controlled touchable motion stimuli with randomizing stimulus trajectories and varying the size. We found a new perceptual illusion in which the motion direction of stimuli perceived on the arm is rotated regionally, or even flipped. In particular, obliquely moving stimuli that move toward the distal radial are perceived as move toward the proximal radial, and stimuli that move toward the proximal ulnar are perceived as move toward the distal ulnar. This illusion was not observed on the palm, regardless of compensation for the stimulus size. Current study adds a clear example of how presenting the same motion stimuli to different body parts results in a different perception, emphasizing that the perceived tactile space is not uniform and needs to be examined in detail.

## Introduction

The tactile perception of motion provides critical information of what we touch and how to react. Given that mechanoreceptors on the skin are heterogeneously distributed and that sampling of neural signals may differ depending on the body part, it must be challenging for the brain to consistently represent motion, for which the integration of spatio-temporal inputs with reasonable speed and accuracy is essential. One effective way to cope with the situation wherein there are inputs of different resolutions from different parts of the body is to change the information processing method according to the site of the input. This has been intensively studied in visual motion research as the contrast between central vision and peripheral vision^1–3^. In the case of touch, on the other hand, such site-specific differences in motion perception have not been subject to extensive scientific scrutiny^4^.

In this study, we will focus on the differences in motion perception between different parts of the body by contrasting the hand and the arm. Motion detection plays a very basic function even at the arm, as described in the previous study^5^ as follows: *“Cutaneous mechanoreceptors of the arm and leg are often primarily thought of as sources of warning signals of the site and direction of motion of stimuli (e.g. a crawling insect) upon the skin surface and of guidance cues for reaching movements (to brush away the insect).”* Not many studies on the arm have been conducted, but some reported the deviation of the spatial representation of the stimulus on the arm from the actual stimulus^4,6–9^. Although such deviation from the Euclidean metric of stimulus representation has been also reported for the hand^10–14^, they differ in occurrence probability and degree from those on the arm. Most previous studies explained their findings by differences in the resolution (receptor distribution and receptive field size)^5,6,12,14^ in addition to the attentional effect^15^.

We have previously reported an error in motion perception at the arm that is difficult to explain solely by the size of the receptive field^4^. When participants touched the braille display with their arms and perceived stimuli presented in a straight path, the perceived angle was found to be distorted only when the stimuli were presented along a specific path. Namely, an inwardly inclined path on the arm was perceived as excessively inwardly inclined, while an outwardly inclined path was perceived accurately. Indeed, this illusion was observed only with the arms but not with the palms. Two important questions remain. The first question is whether this illusion occurs on the arm in general or requires stimulation of a specific skin area of the arm skin. Since the stimulus was presented to a fixed location on the arm in the previous study^4^, the location on the skin subjected to stimulation was slightly different when a right oblique path was presented and when a left oblique path was presented. Thus, the possibility that the involvement of a specific skin area (e.g., close to the thumb) triggered the observed illusion cannot be excluded. The second question is whether this illusion occurs specific to the arms or whether it occurs due to low resolution. The low resolution of the arm cannot explain why the illusion occurs at specific angle and not the other. However, there remains still a possibility that this illusion can be produced anywhere on the body, not just on the arm, by reducing the stimulus clarity, i.e., reducing the size of the stimulus.

Here, we tested to ascertain whether the perceived motion was different when the motion stimulus was presented to the arm versus the hand, irrespective of the local skin site receiving the stimulus and the stimulus size. Participants were asked to report the direction of movement for a diagonally moving dot stimulus, where the initial position of the stimulus was randomized within the stimulus presentation area. Since many previous studies suggested there was a difference in spatial perception between that along the long axis and that along the short axis of the hand and the arm^e.g.,^^7,14^, we measured the perceived direction in each axis as a proportion using two two-alternative forced choices (2 2-AFCs): up/down and left/right. The stimuli were presented to the palm as a standard condition and to the arm as a target condition, with two additional conditions of different stimulus size. One condition was to present a small stimulus to the palm to compensate for the difference in resolution between the arm and the palm. The other condition was to present the stimuli extended along the long axis of the arm to compensate for the perceived length of the stimuli on the arm being shorter for the long axis than for the short axis^5,9^. This anisotropy with regard to the perceived length has been explained by the neurophysiological finding that the receptive field has oval shape^16,17^. Results showed that the accuracy of long-axis direction judgments drops specifically when the motion was presented on an inwardly inclined path to the arm. This trend was not observed when detecting motion on the hand, regardless of compensation for the stimulus size. Current findings add strong evidence that there are body part specific differences in motion perception in touch.

## Results

### Experiment 1

**Figure 1:**
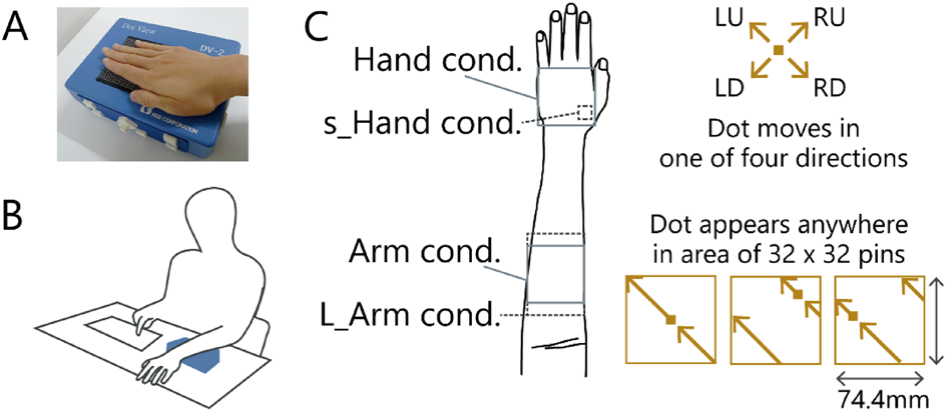
(**A**) Braille-type stimulator. (**B**) View of the setup used in the experiments. The blue box represents the braille stimulator. The participants’ view was blocked by an occluder (not illustrated) and the stimuli were not visible. (**C**) The participants reported the perceived direction of the stimulus trajectory by pressing two arrow keys (e.g., ‘up’ and ‘left’ for the illustrated LU stimuli at the bottom of the diagram).

The purpose of Experiment 1 was to identify the differences in direction discrimination performances depending on (1) the body part to which the stimulus was presented and (2) the direction of the presented motion. The experiment was carried out using the same apparatus (Fig. 1A) and in the same posture as in the previous study^4^. Participants positioned their left hand or forearm on a desk with the palm down (Fig. 1B). This position is natural and is similar to the posture normally used when typing. The stimulus was a dot moving in a diagonal direction (go up the left (LU), go down the left (LD), go down the right (RD), and go up the right (RU) in Fig. 1C) at 50 mm/s for two seconds. Note that the starting point of the stimulus was randomly chosen within the stimulus area in every trial, and the length of the motion trajectory was longer than the two-point discrimination threshold for the forearm^18^.

Participants were first asked (Q1) to report the perceived motion direction of the stimuli, up (distal) or down (proximal) by pressing an arrow key. Then they were asked (Q2) to report the perceived motion direction left (ulnar) or right (radial). (e.g., if the stimulus was LU, the correct response was to press the up and left keys). For each stimulus direction (LU, LD, RD, RU), each question (Q1, Q2), and each body part condition (hand, arm, s_hand, L_arm), the response was calculated for each participant and then averaged across participants.

**Figure 2:**
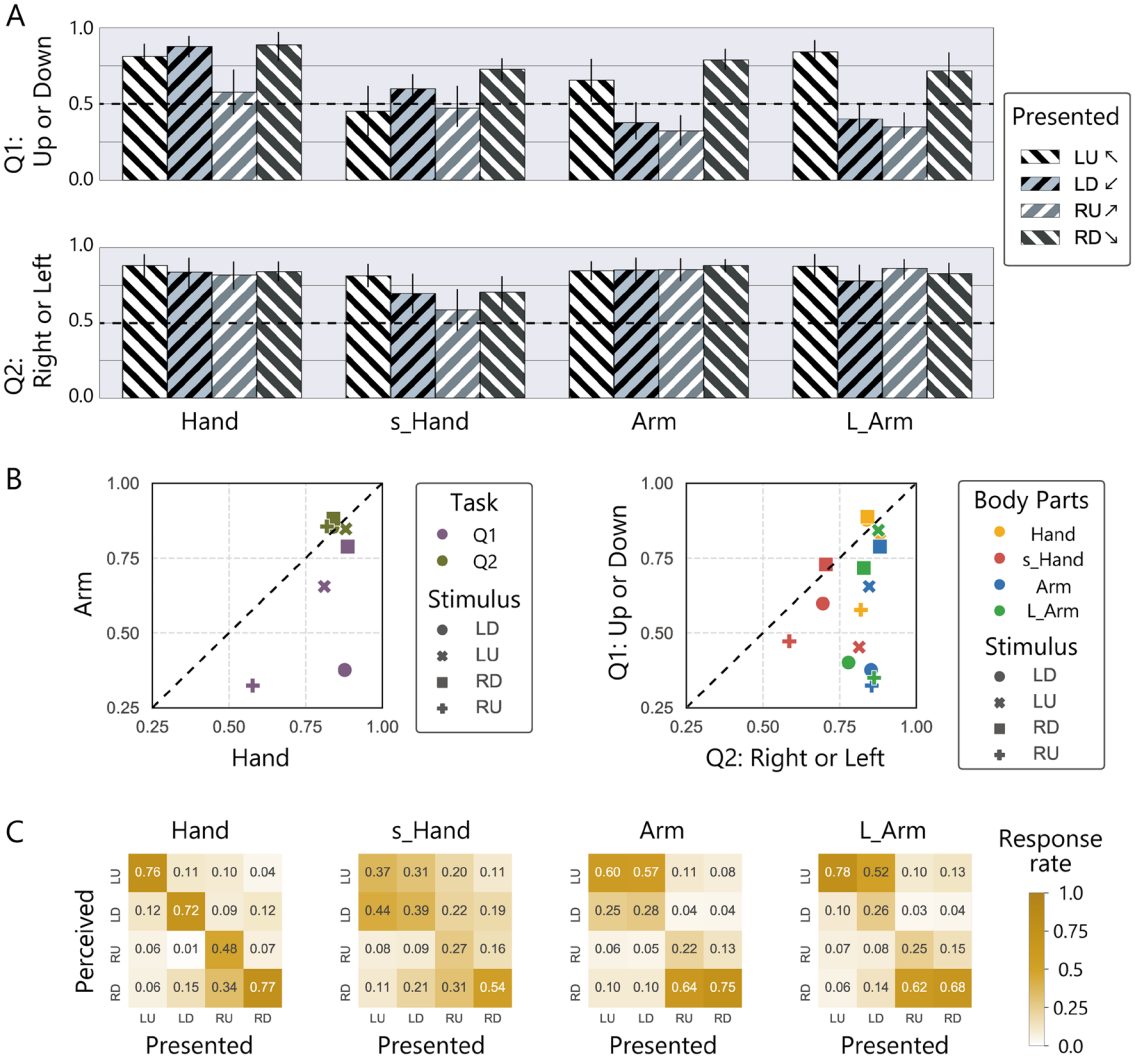
Results of Experiment 1. (**A**) Averaged correct rates for each 2-AFC reported by 12 participants. Error bars represent 95% confidence intervals (CIs). (**B**) Left panel represents the performance relationship between the hand and arm conditions. The vertical axis represents the averaged correct rate for the arm condition, and the horizontal axis represents that for the hand condition. The right panel represents the performance relationship between the two 2-AFCs. The vertical axis represents the averaged correct rate for the vertical 2-AFC, and the horizontal axis represents that for the horizontal 2-AFC. (**C**) The average rate of the perceived direction, estimated from the results of Q1 and Q2. In the confusion matrices, each column represents the presented direction of the stimuli and each row represents the reported direction. Diagonal lines represent correct responses.

The results showed very different trend for the vertical judgment task (Q1) and the horizontal judgment task (Q2), in addition to the performance difference between body parts (Fig. 2A). In Q1, the arm condition showed a clear trend toward lower percentages of correct responses for movement from upper right to lower left (LD) and from lower left to upper right (RU). In other words, the performance was poor when the stimulus was presented on an inwardly inclined path, compared to when it was presented on an outwardly inclined path. In the s_hand condition, the stimulus area was reduced to one-quarter of that under the hand/arm condition to compensate for the difference in two-point discrimination thresholds for the palm and the forearm^18^. Overall performance dropped in this condition, but the observed variation in performance with stimulus direction was different from that under the arm condition. In the L_arm condition, the stimulus area was elongated only in the long axis, taking into account that previous studies reported that distances feel longer along the short axis than along the long axis of the arm^5–7,9^. The degree of elongation along the long axis was 1.4-fold, which was the maximum distance that allowed the arm to make stable contact with the stimulator, a ratio comparable to the perceived space elongation reported for the arm dorsum of human subjects^5^. Performance under this condition showed a similar non-uniform response pattern to that under the arm condition, regardless of the difference in the stimulus area to that of the arm condition.

Full-factorial ANOVA with the GLMM model showed that all main effects and interactions for the fixed effects {body part (4 types), stimulus direction (4 types), and question (2 types)} were significant. For the sake of brevity, we refrain from considering all these effects in detail. Since it has already been reported that there is a difference between the arm and the hand condition^4^, here we report the partial analyses which highlight the new finding of this study, namely, the difference in performance depending on the direction of the stimuli. To explore the significant interaction of questions and other factors, we conducted separate ANOVAs with the GLMM models on Q1 and Q2.

The judgement accuracy in the vertical direction (Q1) varied significantly both with body part (*χ*^2^(3) = 100, *p* < 0.0001) and stimulus direction (*χ*^2^(3) = 54, *p* < 0.0001). Critically, a significant interaction between body part and stimulus direction was observed (*χ*^2^(9) = 160, *p* < 0.0001). The multiple comparison test for the stimulus direction at each body part condition showed that performance with the RU stimuli was significantly lower than that with other stimuli for the hand condition (RU < LU, LD, RD); performance with LU and RU stimuli was significantly lower than that with RD stimuli in the s_hand condition (LU, RU < RD); performance with LD and RU stimuli was significantly lower than that with other stimuli in the arm and L_arm conditions (LD, RU < LU, RD).

The judgement accuracy in the horizontal direction (Q2) showed a different trend: it varied significantly with the body parts (*χ*^2^(3) = 20, *p* < 0.0001) but not with the stimulus direction (*χ*^2^(3) = 0.71, *p* = 0.87). A significant interaction between body part and stimulus direction was observed (*χ*^2^(9) = 27, *p* < 0.01). However, the multiple comparison test for the stimulus direction at each body part condition showed no difference except for the s-hand condition (RU < LU).

These statistical tests suggested that direction discrimination performance was different when the same moving stimulus was presented to the arm and to the hand. More specifically, the results successfully replicate the previously reported phenomena^4^ that the motion perception of a specific direction (LD and RU) was distorted on the arm, even when the stimulating skin area was randomized and when the resolution was corrected. What is more, it was discovered that this distortion occurred only for judgments in the vertical direction, not for judgements in the horizontal direction. In addition, the magnitude of the distortion was the same whether the stimuli move toward (LD) or away (RU) from the body.

Figure 2B shows a scatter plot for the performance of Q1 and Q2. As shown in the left panel, a difference in performance between the hand and arm conditions is evident with Q1 (vertical 2-AFC) while not with Q2 (horizontal 2-AFC). As shown in right panel, the performance of the Q2 task was always better than the level of chance (0.5) except for the s_hand condition where stimuli were presented within a small area. The performance of the Q1 task was generally not as good as the Q2 task, especially for the arm and L_arm conditions with LD and RU stimuli.

Based on the participants’ responses, we drew the confusion matrices showing the relationship between the presented and reported directions of the stimuli (Fig. 2C). In the hand condition, the actual direction of the presented stimulus was most frequently reported, although the correct rate with RU stimuli was relatively low; in the arm condition, the actual direction of the presented stimulus was not necessarily selected most frequently. For example, RU stimuli were reported as RD stimuli in 64% of trials. Clearly, not only did the arm condition show an overall decrease in performance compared to the hand condition, but there was also a tendency for movement from upper right to lower left (LD) and from lower left to upper right (RU) to be reported correctly less frequently. In other words, here we observed a new perceptual illusion in which the motion direction of stimuli perceived on the arm is rotated regionally, or even flipped. Note that it is unlikely that the observed pattern simply reflects a bias in response such that certain keys were pressed more often, since the participants pressed "L" and "R" and "U" and "D" with almost the same probability (e.g., 49% L, 50% R, 46% U, 54% D in the arm condition). To further investigate the effect of response bias, we conducted an additional experiment in which the task was slightly modified, and the illusion was well reproduced, suggesting that the effect of response bias on this illusion is minor (see Supplemental).

### Experiment 2

The purpose of Experiment 2 was to investigate whether and how the attentional demand affects perceived direction of the moving stimuli. In particular, we controlled the trackability of the stimuli to test whether it affects the difference in distortion of direction perception when moving stimuli are presented to the arm and to the hand. In this experiment, nine dots were presented and moved in the same direction. Each of the dots appeared at a random position and moved. Since the stimuli were spatially distributed, it became difficult to focus on a single dot and extract and track its location with attention. This stimulus manipulation may make stimulus tracking with attention difficult. So, tracking of this stimulus might be difficult even when the stimulus is presented to the hand.

**Figure 3:**
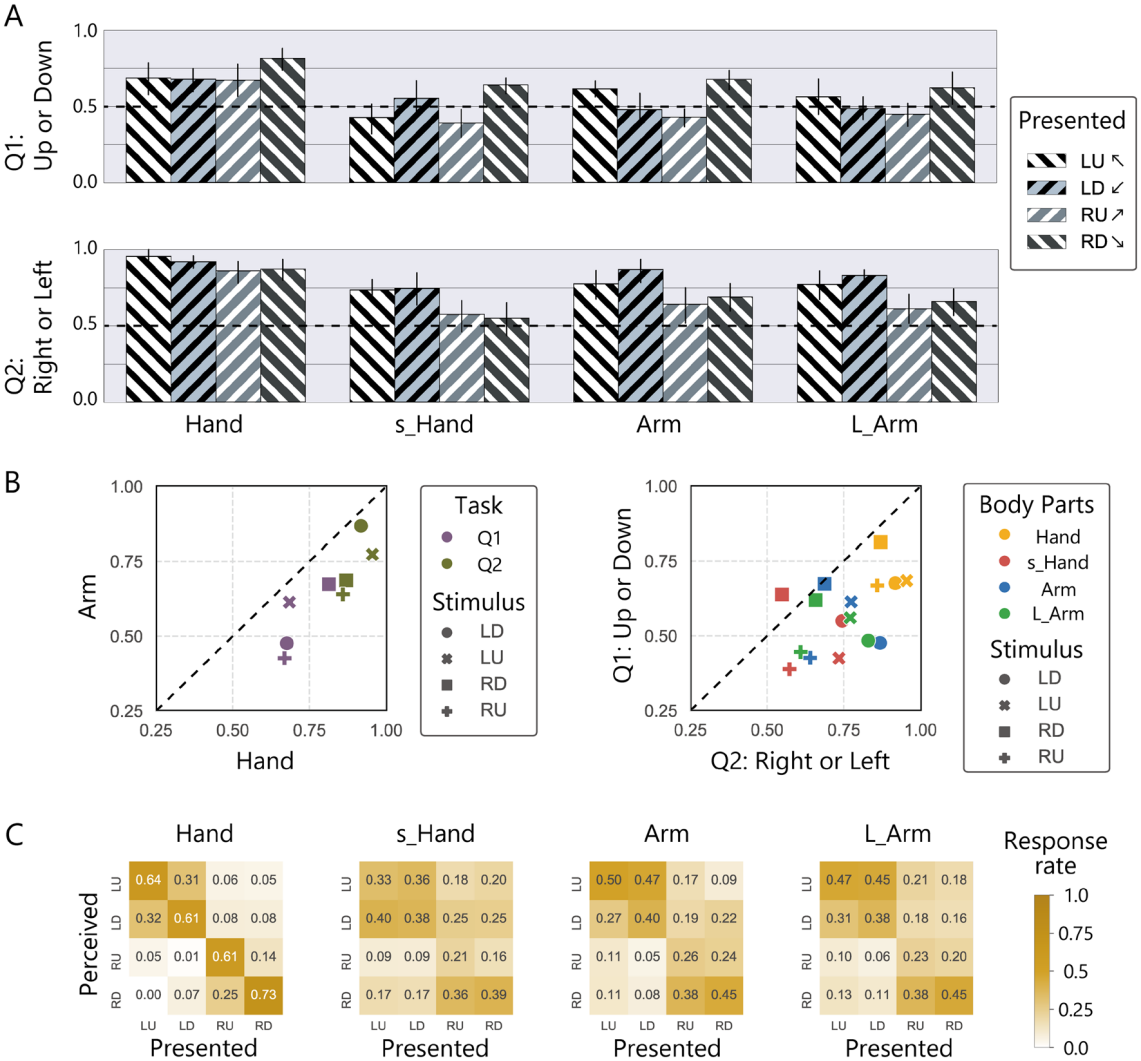
Results of Experiment 2 where nine scatter dots were presented and moved in the same direction. (**A**) Averaged correct rates for each 2-AFC. Error bars represent 95% CIs. (**B**) The left panel represents the relationship of performance between the hand and arm conditions. The right panel represents the relationship of performance between the two 2-AFCs. (**C**) The estimated rate of the perceived direction.

The results in Fig. 3 show an overall drop in performance in most conditions, except for conditions that had already been subjected to the floor effect (around chance level 0.5). As a result, the variations in performance between the hand and arm conditions due to stimulus direction became smaller (Data points are distributed near the diagonal line in Fig. 3B, left panel).

We conducted the same statistical analysis as in Experiment 1, but obtained different results. Two-way ANOVA for the vertical direction (Q1) showed that the judgement accuracy varied significantly with body part (*χ*^2^(3) = 22, *p* < 0.0001), stimulus direction (*χ*^2^(3) = 23, *p* < 0.0001), and the interaction (*χ*^2^(3) = 24, *p* < 0.01). The multiple comparison test showed that characteristic performance bias observed in Experiment 1 (LD, RU < LU, RD) was partly disappeared with the arm condition (LD < RD; RU < LU, RD) and the L-arm condition (RU < RD). Importantly, neither the hand condition (LU, RU, LD < RD) nor the s_hand condition (LU, RU < RD; RU < LU) showed the characteristic performance bias that was observed in the arm condition in Experiment 1. Two-way ANOVA for the horizontal direction (Q2) also showed different pattern from Experiment 1. The judgement accuracy varied significantly both with body part (*χ*^2^(3) = 22, *p* < 0.0001) and stimulus direction(*χ*^2^(3) = 23, *p* < 0.0001), with no significant interaction(*χ*^2^(3) = 14, *p* = 0.13). The multiple comparison test for the body parts showed that performance with the hand condition was significantly higher than other conditions, and that with the arm condition was significantly higher than the s_hand condition. The multiple comparison test for the stimulus direction showed that performance with LD stimuli was significantly higher than RD and RU (RU, RD < LD). It is important to note that the trend observed here is neither the same as that in the arm condition nor that in the hand condition with one dot stimuli (Experiment 1). That is, when stimuli that are difficult to track were presented to the palm, the reported direction bias was not the same as for the arm in Experiment 1.

### Experiment 3

The purpose of Experiment 3 was same to that of Experiment 2: to investigate whether and how the attentional demand affects perceived direction of the moving stimuli, especially its effect on anisotropic distortions of perceived direction of the moving stimuli on the arm. In this experiment, the nine dots moved in a grouped manner like single large dot. The apparent size of the stimulus became larger so the level of difficulty of attentional tracking should be equal to or lower than that for the one-dot condition in Experiment 1 or the nine-scatter-dots condition in Experiment 2. This stimulus manipulation may have facilitated tracking of the stimulus location by attention even when the stimuli were presented to the arm.

**Figure 4:**
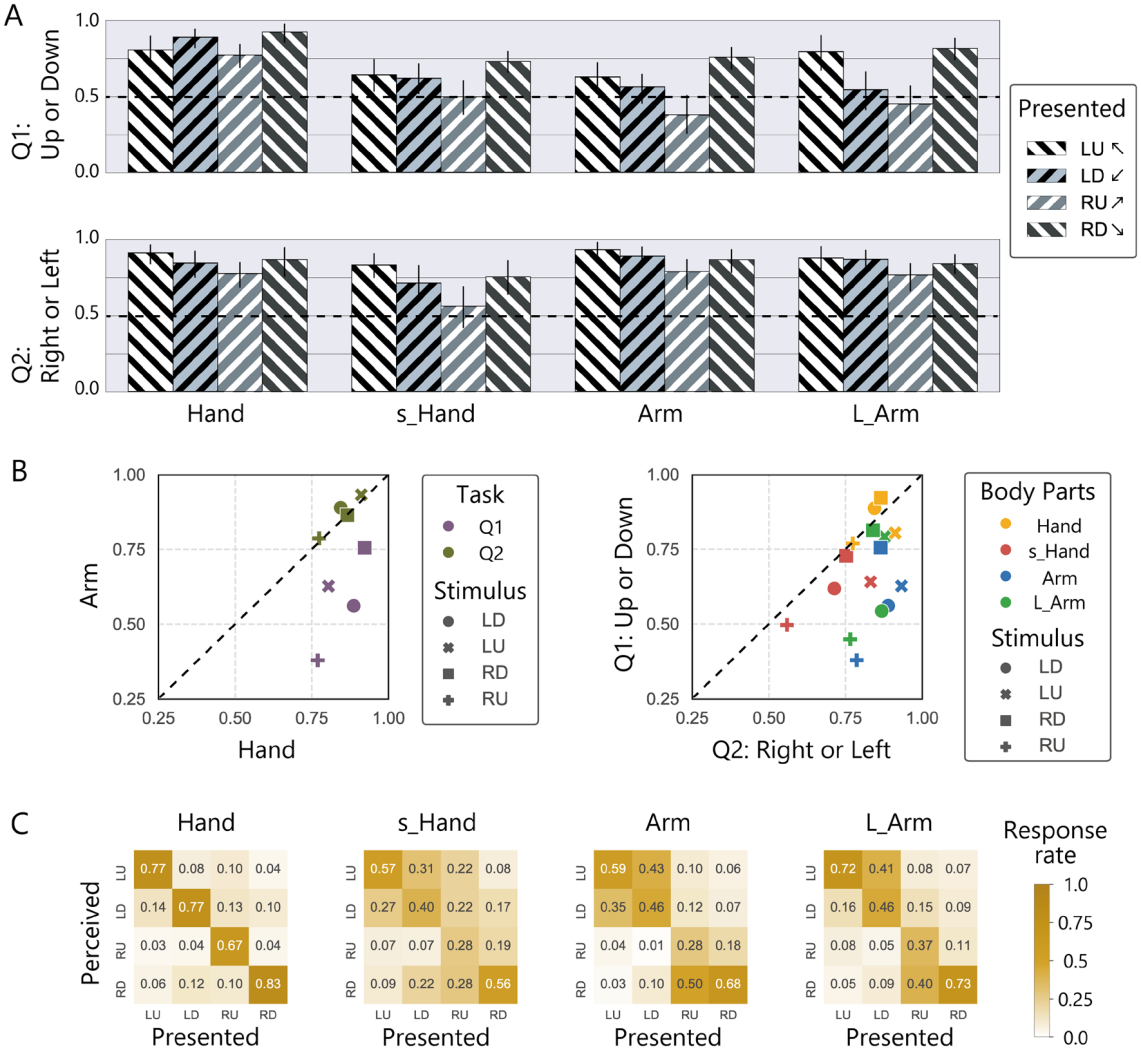
Results of Experiment 3 where nine dots were presented in a grouped manner like single large dot. (**A**) Averaged correct rates for each 2-AFC. Error bars represent 95% CIs. (**B**) The left panel represents the relationship of performance between the hand and arm conditions. The right panel represents the relationship of performance between the two 2-AFCs. (**C**) The estimated rate of the perceived direction.

The results with gathered dots are shown in Fig. 4, showing overall performance improvement in most conditions compared to that with one dot stimuli. Nevertheless, it seems that the characteristic pattern of the correct rates for the vertical judgment task due to the stimulus direction observed in Experiment 1 was replicated for the two arm conditions in this experiment (Fig. 4A). The performance of two arm conditions was still low when the stimulus was presented on an inwardly inclined path, compared to when it was presented on an outwardly inclined path. Accordingly, a scatter plot (Fig. 4B) and the estimated confusion matrices of the relationship between the presented and reported directions of the stimuli (Fig. 4C) show similar patterns to those observed in Experiment 1.

Statistical analysis of Experiment 3 showed a different trend from Experiment 1 or Experiment 2, even though the apparent number of dots or the total area of inputs were the same for these experiments. Two-way ANOVA for each question showed significant interaction of body part and stimulus direction for vertical judgment (Q1, *χ*^2^(9) = 48, *p* < 0.0001) while not for horizontal judgment (Q2, *χ*^2^(9) = 8.0, *p* = 0.54). The multiple comparison test for Q1 showed that characteristic performance bias observed in Experiment 1 (LD, RU < LU, RD) was retained for the L-arm condition, while partly disappeared with the arm condition (LD < RD; RU < LU, RD). In addition, bias in responses also existed in the hand (LU < RD; RU < LD, RD) and s-hand condition (RU < RD), but the trend was different from that of the arm conditions.

Even when stimuli that were easier to track were presented to the arm, performance remained poor compared to the hand and the directional bias did not disappear. Overall, these results of Experiment 2 or Experiment 3 did not support the view that the observed illusion can be explained exclusively by an attentional mechanism.

## Discussion

A series of experiments reported in this paper substantiate the conclusion advanced in our previous report^4^ that there were differences in direction discrimination performance depending on the body part. Participants had difficulty reporting the direction of the moving dot stimuli on the volar surface of the arm when the stimuli were on the inwardly inclined path, but not when the stimuli were on the outwardly inclined path. In addition to findings consistent with the previously reported phenomena that the perception of a specific trajectory was distorted on the arm while not on the palm^4^, three additional findings emerged. First, this bias was clearly observed when the trajectory of the stimuli (i.e., the specific location on the skin where the stimuli were actually presented) was changed, provided the inward tilt of the trajectory was maintained. Second, it was evident that participants had significant difficulty in making longitudinal motion direction judgments (distal vs proximal) with stimuli on the inwardly inclined path compared to stimuli on the outwardly inclined path. The difficulty in making lateral judgments (radial vs ulnar) did not differ significantly regardless of the stimulus trajectory. To the best of our knowledge, this is the first report of a confusion of perceived direction in a specific axis caused solely by a specific angular stimulus. Third, simple compensation for the receptive field size of the hand compared to the arm failed to replicate this illusion on the palm. This finding suggests that this illusion appears only on the arms, not on the hands.

Numerous studies have reported anisotropic distortion in the perceived space in relation to touch^6–14,19–21^. Almost all of the previous studies have discussed this point by examining the difference in perceived size, distance, and location when the stimuli were presented along the long axis and along the short axis. In general, tactile space across the short axis of the body is perceived as larger than space along the long axis, with a substantial difference in the magnitude of such distortion across the body part. The current study was an advance on these previous studies in two principal ways: We used the braille stimulator and presented stimuli on oblique trajectories. The use of the stimulator made it possible to easily change the number and size of stimuli, and what is more, randomize the local skin locations to be stimulated from trial to trial. This type of stimulus manipulation is extremely useful for investigating which parameters are responsible for the observed illusion. Next, we conducted the experiment by presenting stimuli on oblique trajectories instead of cardinal ones, which were mainly used in other studies on tactile spatial distortion. This is because it is well known that stimuli on an oblique angle are perceptually ambiguous compared to stimuli on a cardinal angle^22–26^. Thanks to the high ambiguity of the stimuli, we were able to discover a new illusion in the sense that the illusion is more locally distorted than previously reported illusions of tactile perceptual space^4,6,7,9^: the reported proximal–distal direction (comparison along the long axis) was even “flipped” when the stimuli were presented on inwardly inclined trajectories, but not when presented on outwardly inclined trajectories. This asymmetry was not observed with the reported radial-ulnar direction (comparison along the short axis). The lack of a consistent trend in the variance of proximal–distal direction judgments according to stimulus trajectories in the arm condition (0.66 ± 0.065 for LU, 0.79 ± 0.016 for RD (outward path); 0.38 ± 0.048 for LD, 0.32 ± 0.035 for RU (inward path), see Fig. 2A) suggests that the observed asymmetry is an illusion rather than an increased sensitivity to tactile movements in a certain direction.

We set experimental conditions designed to clarify the involvement of a couple of possibilities that could have an effect on illusions. The first possibility was that this illusion was caused by stimulation of a specific skin location. This point remained an issue needing elucidation due to the inadequacy of the experimental conditions in the previous study^4^, which is the direct motivation of the present study. The second possibility was that the illusion was caused by physiological distortion in the periphery, such as the shape and distribution of receptive fields and skin dermatomes. There are two aspects to this: the simple difference in sensory resolution between the hand and the arm, and the longitudinal and lateral asymmetry that is prominent in the receptive fields of the arm. The last possibility was that the illusion was caused by the mechanism of attention at the level of the central nervous system. These possibilities were not fully clarified by the experiments conducted as part of the previous study^4^, but were thoroughly discussed elsewhere. Each point is discussed in the following sections.

### Effects of skin in specific locations

It may be suspected that the illusion is an inevitable consequence of trivial local differences in skin sensitivity. Indeed, one study reported the difference in sensitivity due to the skin site on the arm–vibration intensity discrimination was better for the thumb side than for the little finger side^27^. Other studies reported differences in localization acuity at different locations along the arm^5,8^. This point was not verified in the previous study^4^, where the stimulus angle uniquely determined which skin site the stimulus would be presented to (e.g., inwardly inclined stimuli always stimulated the local skin site relatively close to the thumb, while outwardly inclined stimuli stimulated the local skin site near the little finger). Such local differences in skin sensitivity, if any, would directly affect the performance of motion detection for a particular angle of stimuli. In this study, we randomized the specific location of the stimuli to tackle this issue, and successfully replicated an illusion similar to that of the previous study^4^. The current finding clearly rules out the possibility that a specific skin site (e.g., the area close to the thumb) explains the distortion of perceived direction of inwardly inclined stimuli on the arm.

### Effects of size difference of receptive fields

Since sensory resolution is markedly different between the hand and the arm^18^, comparing perceived direction under these two conditions was like comparing conditions where the stimulus is viewed with different visual acuity. To compensate for the difference in resolution, we conducted an experiment under the s_hand condition where 1/4 scale stimuli were presented to the palm. If the perception of motion direction in the arm is distorted under certain conditions due to the low sensory resolution of the arm, then the same distortion should be observed when resolution-matched stimuli were presented to the hand. Results showed that the direction discrimination performance naturally decreased under the condition where small stimuli were presented, so the performance under the s_hand condition was closer to that under the arm conditions on average. Importantly, we could not reproduce the characteristics of the illusion, which was the inability to perform a specific task (vertical judgment task) when stimuli at a specific angle (inwardly inclined stimuli) were presented. We conclude that it is difficult to explain the difference in perceived motion direction of stimuli on the arm and that on the hand only by the difference in resolution.

### Effects of geometrically simple and coherent stretching of receptive fields

It has been repeatedly shown that the tactile space across the short axis of the body is perceived as larger than the space along the long axis. These phenomena have been explained in relation to the anatomical trends for the receptive fields of mechanoreceptors^5,6,12,14^ and due to longitudinal distribution of dermatomes^19^ along the long axis^16,28–30^, but see also^31^. According to this theory, a pixel model^10^, stimuli presented along the short axis of the arm are more likely to cover multiple receptive fields and thus likely to be perceived as being larger than the same stimuli presented along the long axis. To minimize this asymmetry as much as possible, we elongated the stimulus area 1.4-fold in the longitudinal direction, which was the maximum size that allowed stable contact of the arm with the stimulator used in this study. This degree of modification was insufficient to cancel out the previously reported elongation of the receptive field on the hindlimb of a cat^29^, but was just enough to cancel out perceived location shift on the arm dorsum of human subjects^5^. As a result, the overall trend of motion direction perception in the L_arm condition was not much different from that in the arm condition. In other words, the illusion did not disappear with simple elongation of the stimulus area. It remains to be seen whether size modification at extreme ratios will eliminate the distortion of direction perception on the arm. Still, geometrically simple and coherent stretching of tactile space does not account for the anisotropy that the observed illusion occurs only with inwardly inclined stimuli but not with outwardly inclined stimuli.

### Effects of attention

It has been reported that the perceived location of the input stimulus or perceived tactile space changes according to how the stimulus is applied, even when the same stimulus is applied to the same location of the skin^13,21,32,33^. Such phenomena cannot be explained by anatomical structures such as receptor distribution, and it is natural to assume that this is an effect of central processing. More directly, some studies have reported that top-down and bottom-up attention can affect localization performance. For example, participants were able to localize input stimuli more accurately when they were explicitly instructed to focus their attention on the stimulus site, compared to when they focused their attention on the entire arm^15^.

The observed illusion can be interpreted as reflecting an attentional effect, for example, by making the assumption that inwardly inclining movements are more difficult to track with attention. This explanation requires another assumption to explain why the illusion occurs only on the arms. For example, a possible explanation could be that attentional tracking was more difficult and a drop in performance was evident on the arm because the stimulus is perceived as blurred on the arm but clear on the hand. To control the trackability of the stimuli, we conducted Experiments 2 and 3, in which nine dots moved in the same direction with different spatial distributions. Since the stimuli were spatially distributed in Experiment 2, it can be speculated that it was difficult to focus on a single dot and extract and track its location with attention even when the stimuli were presented to the hand. Conversely, in Experiment 3, the nine dots were spatially grouped and displayed as one large dot, which may have facilitated tracking the stimulus location by attention even when the stimuli were presented to the arm. Here, the difficulty of following the stimuli on the arm becomes less and may resemble the difficulty of following the one-dot stimulus on the hand (Experiment 1). If the attentional effect is the key for this illusion, we expect the illusory distortion of the perceived direction on the arm to be diminished in Experiment 3.

**Figure 5:**
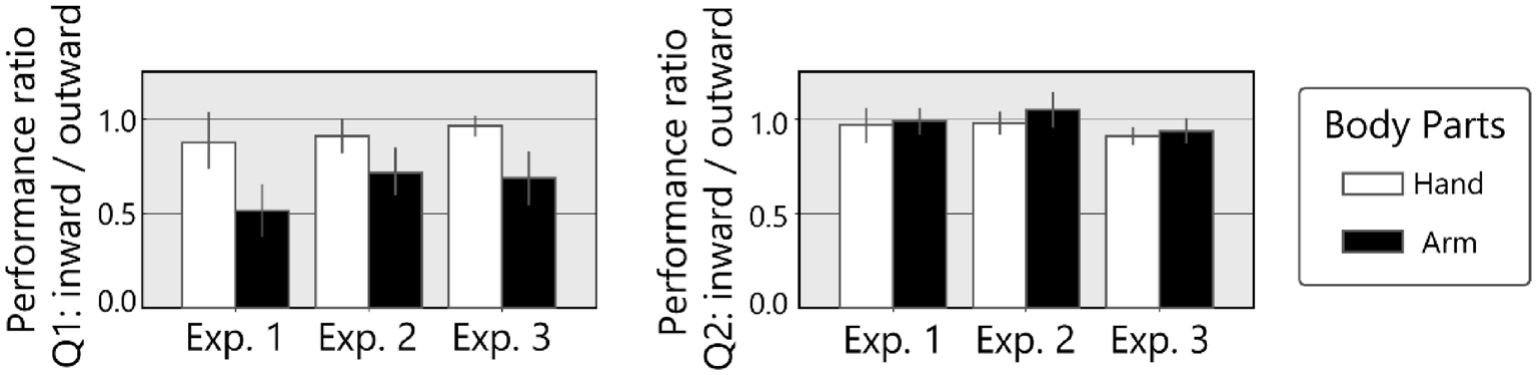
Comparison of performance in direction judgments for different stimulus trajectories. Performance ratio represents the task performance when the stimuli were on the inwardly inclined path (LD and RU) divided by that when the stimuli were on the outwardly inclined path (LU and RD). The left panel shows the ratios for Q1 and the right panel for Q2.

To visualize the intensity of illusion (i.e., difference in the difficulty of directional judgments with the stimuli on the inwardly inclined path or outwardly inclined path) across experiments, we calculated performance ratios. Figure 5 represents the task performance with LD and RU stimuli (inward) divided by that with LU and RD stimuli (outward). The ratio of the hand condition was around 1.0 for vertical judgment (Q1), suggesting asymmetry due to stimulus direction was negligible (no illusion). Meanwhile, the ratio for Q1 was low with the arm, showing performance asymmetry (i.e., the task was more difficult with inward motion stimuli than that with outward motion stimuli). If this body part difference can be explained simply by differences in attentional effects, in particular differences in the difficulty of tracking stimuli, two trends are expected as described above: in Experiment 2, the ratio is low even in the hand condition because the presented stimulus was difficult to track; and in Experiment 3, the ratio is high even in the arm condition because the presented stimulus was large and easy to track. However, such trends were not observed. ANOVA with the GLMM model for the rates of Q1 (Fig. 5, left panel) showed significant differences between body parts (*χ*^2^(1) = 32.0, *p* < 0.0001), while failed to show any significant differences between experiments (*χ*^2^(2) = 5.0, *p* = 0.08) nor the interaction (*χ*^2^(2) = 2.0, *p* = 0.4). In addition, two-way ANOVA for the rates of Q2 did not show any significant difference (*χ*^2^(1) = 3.1, *p* = 0.08 for body part; *χ*^2^(2) = 4.1, *p* = 0.12 for experiment; *χ*^2^(2) = 1.0, *p* = 0.6 for the interaction).

In summary, we have not found any strong evidence to support the hypothesis that the attentional difficulty in following the signal in a particular direction is the main cause of this illusion.

### Limitations and Future Work

In this study, as in the previous study^4^, there are limitations due to the use of the braille display. The limitations are primarily a result of the slow refresh rate and flat display surface. Stimulus speed clearly has an impact on the difficulty of attentional tracking, and it is reasonable to question its effect on the reported illusion. The speed of 50 mm/s used in the present study was originally determined on the basis of our previous study^4^ and preliminary experiments. Due to limitations of the stimulator, we could not test with much faster stimuli. Note however that in the L_arm condition, the stimuli were slightly faster than in the arm condition and a robust illusion was still observed. When stimuli of half the speed were presented, overall task performance decreased and the illusion became weak (see Supplemental Fig. S2). It is not possible to conclude at this point whether the illusion occurs at a specific motion speed and not in the slow stimulus condition, or whether the illusion disappears due to the floor effect because it is difficult to judge the direction of motion when the stimuli are presented at a slow speed. The effect of speed on the illusion awaits further empirical validation with an appropriate stimulator.

Another limitation is a potentially incomplete contact condition. Since the arm is not perfectly cylindrical in shape nor is its fleshy tissue uniform, whereas the stimulator has a flat surface, it is quite possible that the contact between the arm and the stimulator is not physically uniform. To address this, in the previous study^4^, we examined perception while touching the device in several postures, but could not systematically control for contact conditions due to the flat surface. A productive direction for future work would be to develop a device capable of uniformly stimulating heterogeneous points on the skin of the arm.

It was found that the size difference or simple stretching of receptive fields and attentional tracking cannot fully account for this illusion. Several possibilities remain that have not been experimentally considered in this study.

One possible explanation is the difference in the type of mechanoreceptors between the hand and the arm. The palm is covered by glabrous skin and the arm is covered by hairy skin, with different types and distribution of mechanoreceptors. In particular, the detection of low-frequency vibrotactile inputs from the glabrous skin appears to depend on the Meissner corpuscles, while that from the hairy skin depend on the hair follicle receptors^33^. Accordingly, vibrotactile detection thresholds on these two types of skin are very different, while the frequency discrimination performance has been reported to be comparable^33,34^. The detection of high-frequency vibrotactile inputs from two skin sites appears to depend on the same kind of receptor, Pacinian corpuscles, but they are embedded much more deeply in hairy skin^35^. Therefore, it remains an open question if such differences cause body-part-specific illusions.

Another possible explanation is the inwardly inclined and stretched *anisotropic* receptive field on the volar surface of the arm. It has been suggested in previous studies^10,12^ as a pixel model that the key information for computing tactile distance may be the number of receptive fields along the stimulated locations. Based on this model, an anisotropic receptive field fits well with the current illusion, and future detailed neurophysiological research of the arm may provide valuable insight.

An alternative, although not exclusive, possibility is that the receptive field is stretched by rotation of the arm. When the forearm is rotated from palm up to palm down, the skin of the arm deforms accordingly. In this case, the wrist rotates 180° while the elbow rotates through a smaller angle, so the skin and underlying flesh may stretch along a particular axis rather than homogenously. This may cause a pseudo diagonal oval shape of the receptive field on the arm in the palm-down posture. Moreover, this skin anisotropic stretch may cause a mismatch in the frames of reference between the haptic space and the physical space. If humans localize stimuli according to a skin-based reference frame, then anisotropic stretching of that skin would result in physical vertical stimuli not being perceived as vertical. Whether the reference frame of haptic space of the arm is purely based on the skin or a combination of the skin and arm rotation is still under debate^13^. It would also be an interesting angle for future study to validate directional judgments with arms in the palm-up posture using appropriate stimulators.

It is also possible that this illusion reflects some characteristics of central tactile processing. There may be site-specific differences in the temporal integration window and/or in the computation of integrating spatio-temporal information. This hypothesis still awaits further empirical validation. It would also be worthwhile to examine the neural representation, since one study showed representational distortions in S1 that correspond to distortions in distance perception at the periphery^36^. In addition, the biological significance of the observed illusion remains an intriguing open question.

### Implications

A new illusion regarding anisotropic distortion in the perceived direction of motion on the arm is reported. This finding further supports the notion that the perceived tactile space is not uniform, and needs to be examined in detail. For example, there are several recent studies reporting that an anisotropic stretching of perceived space at the lower back was observed in exactly the opposite direction to that at the upper back^21,37^. Further detailed investigations for each body part may reveal new illusions.

## Methods

### Participants

Twenty-two naïve participants (four men; aged 20 to 45 years; mean age 32.8 ± 8.35 years; all right-handed) with normal tactile sensitivity (based on their self-report) participated in the experiments. Twelve participants took part in Experiments 1. Another group of 12 participants participated in Experiments 2 and 3, with partial overlaps of participants across experiments. All gave written informed consent before the start of the experiment. NTT Ethics Committee approved the recruitment of the participants and the experimental procedures, which were conducted in accordance with the ethical standards that have their origin in the Declaration of Helsinki (2008). (Approval number: H28-009, R02-001).

### Apparatus

Tactile stimuli were presented to the participants using the same piezoelectric braille display (stimulator, hereinafter) (Dot View DV-2, KGS, Japan) employed in the previous studies^4,38^. The stimulator has an array of pins with a diameter of 1.3 mm and an inter-pin distance of 2.4 mm. Each pin can be switched independently to either the “on” position (maximum 0.7-mm normal displacement or less when damped by the contacting hand/arm) or the “off” position (no displacement), and the status of the pins (“on” or “off”) was updated every 100 ms. Note that on-pins did not vibrate (which is typical of other braille systems such as OPTACON (Telesensory Systems, Palo Alto, CA)) but remained stationary during the specified period with a rise time of 15 ms.

The stimulus was a dot moving in a diagonal direction (LU, LD, RU, and RD in Fig. 1C) for two seconds. The dot stimulus consisted of four to six “on” pins and moved every 0.1 to 0.2 s. In the s_hand condition, the dots consisted of one to two pins. These variations were unavoidable due to the innate characteristics of the stimulator. The stimuli were presented within 32 × 32 pins (76 mm × 76 mm) at a speed of 50 mm/s for the hand condition and arm condition, while they were presented within 8 × 8 pins (18 mm × 18 mm) at 12 mm/s for the s_hand condition and 45 × 32 pins (107 mm × 76 mm) at 66 mm/s for the L_arm condition. These speeds and duration were adopted following the previous study^4^.

The experimental manipulation of randomizing stimulus location is essential to testing the main hypothesis of this study. If we attempt to present continuous motion for the full stimulus duration in a limited stimulus area, the starting point of the stimulus will be restricted. Then, the starting point of the stimulus itself becomes a cue to determine the direction of motion, which is the same situation as in the previous study^4^. Therefore, here, we decided to split the trajectories of the stimuli if necessary. The starting point of the stimulus was randomly chosen in every trial, and when the stimulus reached the edge of the stimulus area, it appeared from the opposite edge. This repetitive motion could induce ambiguity regarding the direction of motion, although the direction could be easily reported visually (preliminary reports). The length of the trajectory was varied across trials, but in all trials, the dot was moved along the same trajectory for more than half the diagonal of the stimulus area. In the arm and the L_arm condition, this length is longer than the two-point discrimination threshold for the forearm^18^.

The stimulus was one dot in Experiments 1. The stimulus was nine dots moving in the same direction with a random spatial pattern in Experiment 2, and one large dot consisting of the same number of pins as the nine dots was presented in Experiment 3.

### Experimental design

The experimental setup and procedure were almost identical among experiments. Each participant sat at a table and touched the stimulator with the volar surface of their left hand or forearm. The stimulator was located to the left of the body midline so that participants could comfortably place their hand/arm. This posture is the same as that adopted in the previous study^4^ and is commonly used in daily situations such as typing. Contact between the stimulator and the participants’ skin was maintained throughout the experiment. They responded by pressing a keyboard with their right hand. They performed the tasks facing forward with their eyes open to maintain their arousal level. The display parts of the stimulators were occluded from the participants’ view by a blackboard.

There were four different body part conditions, and the body part was fixed during one block. The stimuli were presented to the palm as a standard condition (hand condition) and to the arm as a target condition (arm condition), with two additional conditions of different stimulus size. One condition was to present a small stimulus to the palm to compensate for the difference in resolution between the arm and the palm (s_hand condition). The other condition was to present the stimuli extended along the long axis of the arm (L_arm condition) to compensate for the perceived length of the stimuli on the arm being shorter for the long axis than for the short axis^9^. Note that the spatial relationship between the presented stimulus (i.e., the top surface of the stimulator) and the participant's body (eyes, torso) was fixed and was the same for all body part conditions for the same participant. At the beginning of each block, the braille stimulator presented a square or rectangular shape to remind participants of the presentation area of the stimuli. In the hand condition, the participant was instructed to touch the stimulus presentation area with their left palm. In the s_hand condition, the participant was instructed to touch the stimulator with their left thenar area. In arm and L_arm conditions, the participant was instructed to touch the stimulator with the volar surface of their left forearm. The participants were asked to press a key when they were ready. After the key press, all the pins went into the off position and a beep sounded to signal the start of the trial. After a 500-ms blank period, dot stimuli appeared at a random location in the stimulus presentation area and started to move in one of four diagonal directions. After presenting stimuli for 2,000 ms, all the pins went into the off position.

In the experiments, the participants were first asked to report the perceived motion direction of the stimuli, up (distal) or down (proximal) by pressing the arrow key as in Q1. Then they were asked to report perceived motion direction left (ulnar) or right (radial) as in Q2. (e.g., if the stimulus was RD, press the down and right keys). After each key press, the feedback signal was provided as a beep sound when the response was incorrect. After a 500-ms blank period, the next trial started. Each block lasted around five to ten minutes.

Before the experiment, the participants conducted a practice session in which they familiarized themselves with the stimuli and the task by looking at and touching the stimuli and by pressing keys to report the perceived direction. Each participant performed trials consisting of 4 body part × 4 direction of motion × 8 ~ 12 repetitions for each experiment. The order of the trials was pseudo-randomized for a participant and also varied across the participants. The total time including practice and breaks for each participant for each experiment was around two hours. A trial was excluded from later analysis when participants reported they failed to detect a signal (not discrimination) (e.g., due to poor hand placement).

Bar graphs show the averaged data across participants, with 95% CIs calculated using a bootstrapping method^39^. We analysed all individual data for perceived direction in each experiment (whether the reported direction was correct or wrong for each question on each trial in each stimulus condition) using the Binomial generalized linear mixed model (GLMM)^40^. In the discussion, performance ratios were calculated to compare when stimuli were presented on inward or outward path. These ratios were analysed using the Poisson GLMM. We first made a statistical inference using a maximum model (model with all possible fixed and random effects) and then conducted an automated model selection with backward elimination. Based on the selected model, we implemented a full-factorial analysis of variance (ANOVA) for fixed effects (Wald *χ*^2^ tests). We further performed multiple comparisons with a corrected alpha level (*p* < 0.05) using the Bonferroni method when there was a significant main effect. Analyses were performed using R with *lme4*, *multicomp*, *lsmeans*, and *buildmer* package.

## Supplementary Information

Supplementary Information.

## Data Availability

The datasets used and/or analysed during the current study available from the corresponding author on reasonable request.
