# Supplementary material for: Anisotropic distortion in the perceived direction of motion on the arm
Source: Sci Rep. 2023 Jan 2;13:69. doi: 10.1038/s41598-022-27032-x (PMC9807636; doi:10.1038/s41598-022-27032-x)
Supplement: Supplementary file 1 — Supplementary Information. [file 41598_2022_27032_MOESM1_ESM.pdf]

Supplemental material for

Anisotropic distortion in the perceived direction of motion on the arm

Scinob KUROKI*

* E-mail: scinob@gmail.com

Experiment S1

The purpose of Experiment S1 was to test the possibility that the observed response pattern in Experiment 1 might be simply explained by participants' response bias. Although it is unlikely, there remains a possibility that for no particular reason our participants tended to respond with a combination of right and down keys or left and up keys only when the stimuli were presented to the arm, resulting in a higher probability of answering LU or RD. To rule out this possibility, we slightly modified the task. In this experiment, the same participants as those in Experiment 1 were asked to report the trajectory of the perceived motion (Q1: tilted to the right or to the left) and then report the moving direction of the dot on the trajectory (Q2: up or down). Therefore, the keys expected to be pressed are different in Experiments 1 and S1, even when the same stimuli are presented. For example, for LU stimulus, the correct reports are 'up' and 'left' in Experiment 1 and 'left' and 'up' in Experiment S1; for LD stimulus, the correct reports are 'down' and 'left' in Experiment 1 and 'right' and 'down' in Experiment S1.

The results in Fig. S1 show a similar perceived pattern to that observed in Experiment 1. An analysis of ANOVA with the GLMM model for the rates of Q1 (χ^2 (3) = 78, $p < 0.0001$ for body part; $\chi^2(3) = 46$, $p < 0.0001$ for stimulus direction; $\chi^2(9) = 100$, $p < 0.0001$ for the interaction) and Q2 (χ^2 (3) = 13, p < 0. 01 for body part; $\chi^2(3) = 14$, $p = 0.05$ for stimulus direction; $\chi^2(9) = 40$, $p < 0.0001$ for the interaction) was in good agreement with the analysis of Experiment 1. This result clearly rules out the possibility that observed anisotropic distortion in the perceived direction of motion on the arm simply reflects a bias in pressing keys.

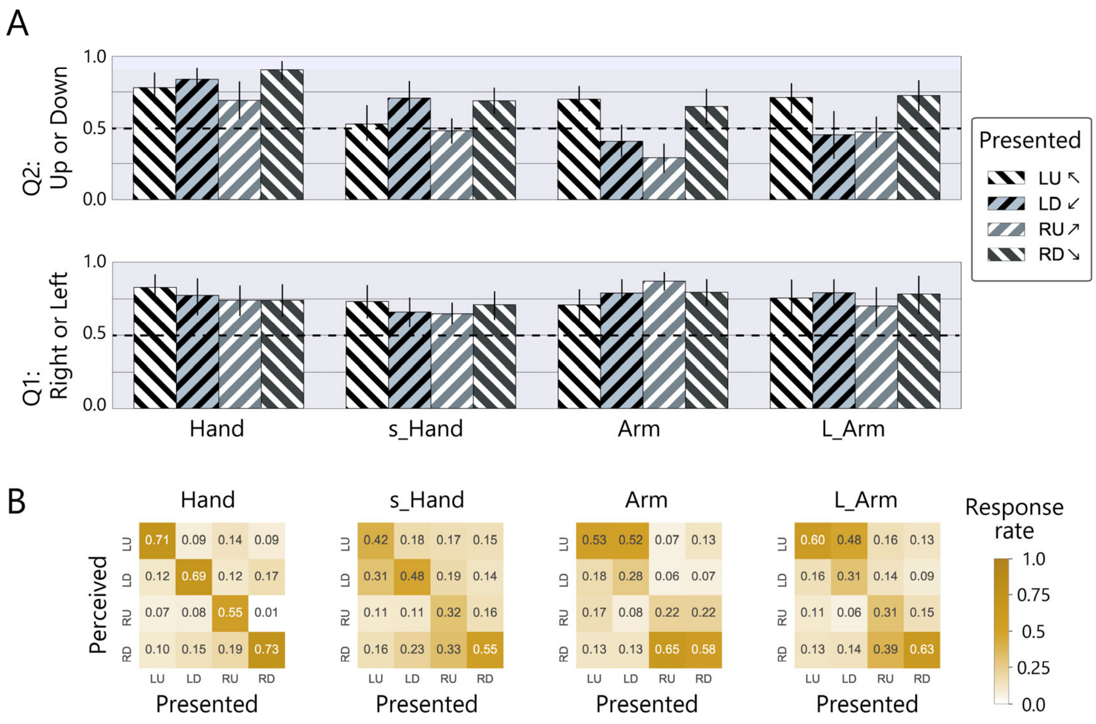

Fig. S1. Results of Experiment S1. (A) Averaged correct rates for each 2-AFC. Error bars represent 95% CIs. (B) The estimated average rate of the perceived direction. Note that what the participants reported in this experiment was the trajectory (Q1) and direction (Q2) of the moving dots, but in this figure, obtained responses are shown converted to the direction of the moving dots corresponding to Fig. 2 (e.g., The report of a rightward tilt trajectory and downward movement was represented as LD (leftward and downward motion) in the figure)

Preliminary experiment S2

As a preliminary experiment S2, we had conducted an experiment with a small number of participants (N=10) with the speed of movement reduced to half that of Experiment 1. The performance in directional judgement decreased in all conditions, and the illusion became weaker (Fig. S2). That is, a significant interaction between body parts and stimulus direction was observed for vertical direction judgment (χ^2 (9) = 34, $p < 0.001$), but the multiple comparison test did not show the characteristic performance bias observed in Experiment 1 (LD, RU < LU, RD). It is not possible to conclude at present whether the illusion occurs at a specific speed of movement and therefore does not occur under slow stimulus conditions, or whether the illusion disappears due to the floor effect because it is difficult to judge the direction of movement with slow stimuli.

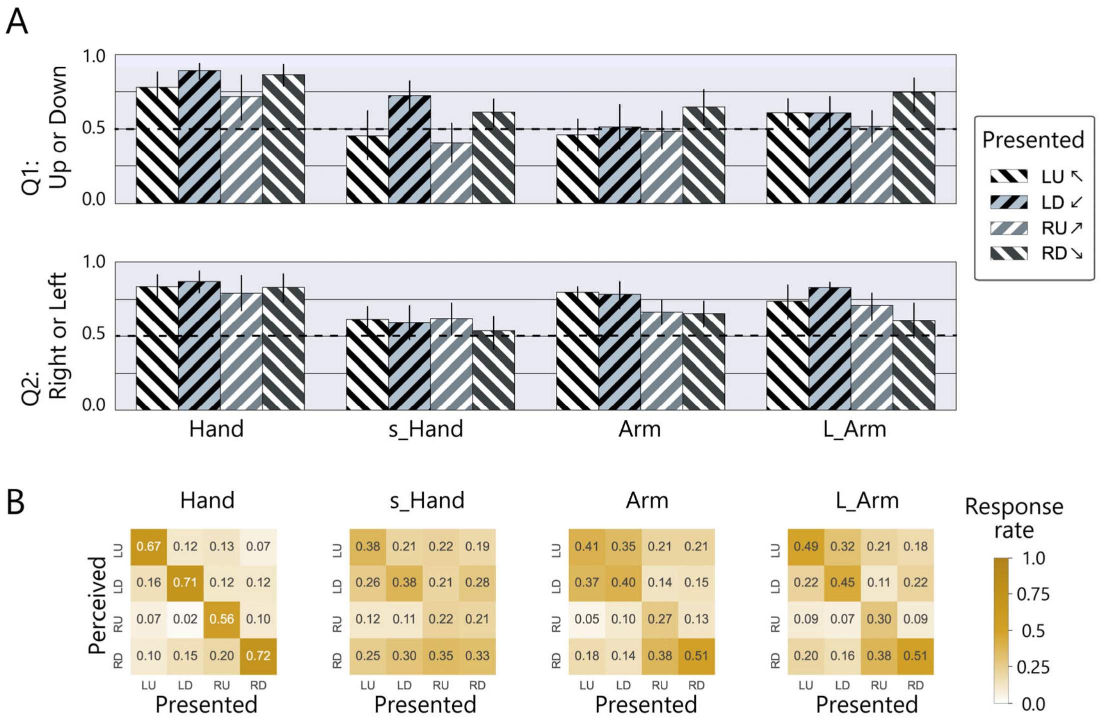

Fig.S2. Results of preliminary experiment with slow speed motion stimulus. The average rate of the perceived direction was reported by 12 participants. In the confusion matrices, each column represents the presented direction of the stimuli and each row represents the reported direction. Compared to the results for the normal speed stimulus in Fig. 2A, the overall performance decreased. As a result, the characteristic response pattern for the arm condition observed in Fig. 2A is obscured in this figure.
